# Cytogenomic characterization of three murine malignant mesothelioma tumor cell lines

**DOI:** 10.1186/s13039-020-00511-4

**Published:** 2020-09-09

**Authors:** Eva Wahlbuhl, Thomas Liehr, Martina Rincic, Shaymaa Azawi

**Affiliations:** 1 Jena University Hospital, Institute of Human Genetics, Friedrich Schiller University, Am Klinikum 1, 07747 Jena, Germany; 2 Croatian Institute for Brain Research, School of Medicine, University of Zagreb, Salata 12, 10000 Zagreb, Croatia

**Keywords:** Murine multicolor banding (mcb), Array comparative genomic hybridization (aCGH), Malignant mesothelioma, Murine cell line, AB1, AB22, AC29

## Abstract

**Background:**

Malignant mesothelioma (MM) is a rare aggressive cancer primary located in pleura and lung. MMs can be divided into biphasic, epithelioid and sarcomatoid subtypes. In majority of cases MMs are induced by asbestos fiber exposure. As latency period after asbestos exposure ranges between ~ 10 and 60 years MMs are mainly observed in elder people. Human MM, being a rare tumor type, lacks detailed cytogenetic data, while molecular genetic studies have been undertaken more frequently. However, murine MM cell lines are also regularly applied to get more insight into MM biology and to test new therapy strategies.

**Results:**

Here the murine MM cell lines AB1, AB22 and AC29 were studied by molecular cytogenetics and molecular karyotyping. Interestingly, yet there were no genetic or genomic studies undertaken for these already in 1992 established cell lines. The obtained data on genomic imbalances in these murine cell lines was translated into the human genome as previously reported based on human and murine genomic browsers.

**Conclusions:**

It turned out that all three cell lines showed high similarities in copy number variants as observed typically in human MM. Also, all three cell lines were most similar to human epithelioid MMs, and should be used as models therefore.

**Electronic supplementary material:**

The online version of this article (10.1186/s13039-020-00511-4) contains supplementary material, which is available to authorized users.

## Background

Malignant mesothelioma (MM) is a rare aggressive tumor-family of pleura and lung, with an incidence of about 0.002% [1, 2]. In most of the cases, MMs are located in pleural mesothelium, and only rarely in peritoneal cavities, tunica vaginalis or pericardium. MM can be specifically promoted by exposure to asbestos fibers [3, 4]. Besides working with asbestos, accordingly contaminated buildings provide an additional, often unrecognized problem, where affected person can undergo asbestos inhalation, ingestion, or less often, severe exposures via the skin [3, 5]. The latency periods for MM after asbestos exposure can range from 1 to 6 decades, and the median age of onset is 72 years [6].

Numerous genetic changes are involved in MM. These include numerical and structural chromosomal aberrations and molecular genetically detectable alterations in the cellular signal transduction pathways, among others caused by activation of oncogenes or loss of tumor suppressor genes [5]. In human the genes cyclin-dependent kinase inhibitor 2A (*CDKN2A*), neurofibromatosis type 2 (*NF2*), the breast cancer associated gene 1 (*BRCA1*) associated protein 1 (*BAP1*) and tumorsuppressorprotein 53 (*TP53*) genes seem to be major players in MM-pathogenesis and -progression [7–16].

Histomorphologically and according to their growth parameters, MM can be divided into the following, most frequently observed subtypes: (1) biphasic, (2) epithelioid and (3) sarcomatoid. Different median survival times were attributed to each subtype; the best prognosis has the epithelioid, while the worst one has the sarcomatoid subtype [13, 17].

As MM is an aggressive tumor with poor prognosis, there is ongoing research to better understand the biology of this cancer type [18, 19]. Therefore, also animal models, including murine tumor cell lines are regularly applied, also because human and mouse genomes show homologies within coding sequences of up to 97% [20]. In 1992 Davis and coworkers inoculated asbestos fibers into female BALB/c and CBA mice and established successfully 12 MM cell lines from tumor ascites cells [21]. Here two of these cell lines derived from BALB/c mice, i.e. AB1 and AB12, and one of them from CBA mice (AC29) were studied. Strikingly, in none of these cell lines (cyto)genetic research was undertaken yet to characterize their cytogenomic content. However, the latter data are important to use such cell lines in the best suited way to answer questions about MM-biology or to apply them in tests for new treatment options, i.e. for drug tests meant for the corresponding MM subtype.

## Results

### Molecular cytogenetics

#### Ab1

**Fig. 1:**
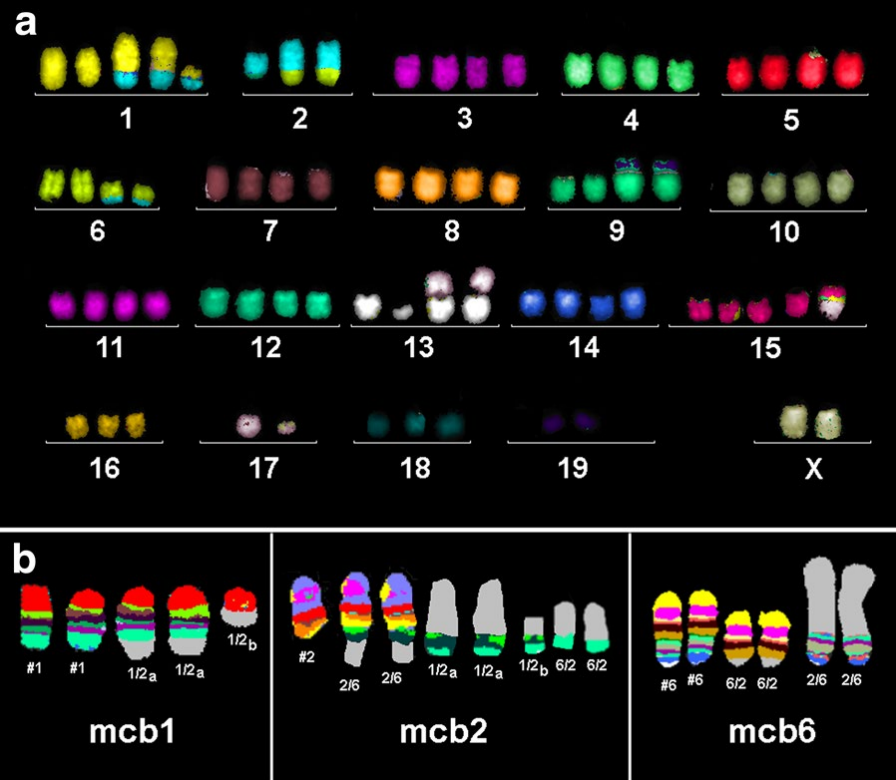
**a** Results of mFISH using all 21 murine whole chromosome paints as probes applied on murine MM cell line AB1 are shown here. **b** Typical pseudocolorbanding-results for murine multicolor banding (mcb) as applied on cell line AB1 for chromosomes 1 (mcb1), 2 (mcb2) and 6 (mcb6). Derivative chromosomes are shown as 1/2_a_ = der(1)t(1;2)(H5;F1), 1/2_b_, = der(1)t(1;2)(C1;F1), 2/6 = der(2)(2A1→2H4::6F3~G1→6E1::6F3~G1→6qter) and 6/2 = der(6)t(2;6)(H1;E1); normal chromosomes are labeled by # and chromosome number

This cell line showed the following hypotetraploid composite karyotype (Fig. 1a) 73~80<4n>,-X,-X,der(1)t(1;2)(H5;F1),der(1)t(1;2)(H5;F1),+der(1)t(1;2)(C1;F1),-2,der(2)(2A1→2H4::6F3~G1→6E1::6F3~G1→6qter),der(2)(2A1→2H4::6F3~G1→6E1::6F3~G1→6qter),der(2)t(2;19)(E3;D1),+3,der(6)t(2;6)(H1;E1),der(6)t(2;6)(H1;E1),der(7)t(7;19)(E3;D1),dic(9;19)(A1;D3),dic(9;19)(A1;D3),del(13)(A5),dic(13;17)(A1;A1),dic(13;17)(A1;A1),der(15)t(15;?)(E1?;?),+der(15)(15pter→15E1::17B→17E3::17E3→17B:),+der(15)(15pter→15B2::17B→17E3::17E3→17B:),-16,-17,-17[11],-18[10],del(19)(D1),del(19)(D1).

In Fig. 1b examples of mcb experiments are shown for chromosomes 1, 2 and 6, which enabled the characterization of the der(1)t(1;2)(C1;F1), the der(1)t(1;2)(H5;F1)x2 and the der(2)(2A1→2H4::6F3~G1→6E1::6F3~G1→6qter)x2.

#### Ab22

**Fig. 2:**
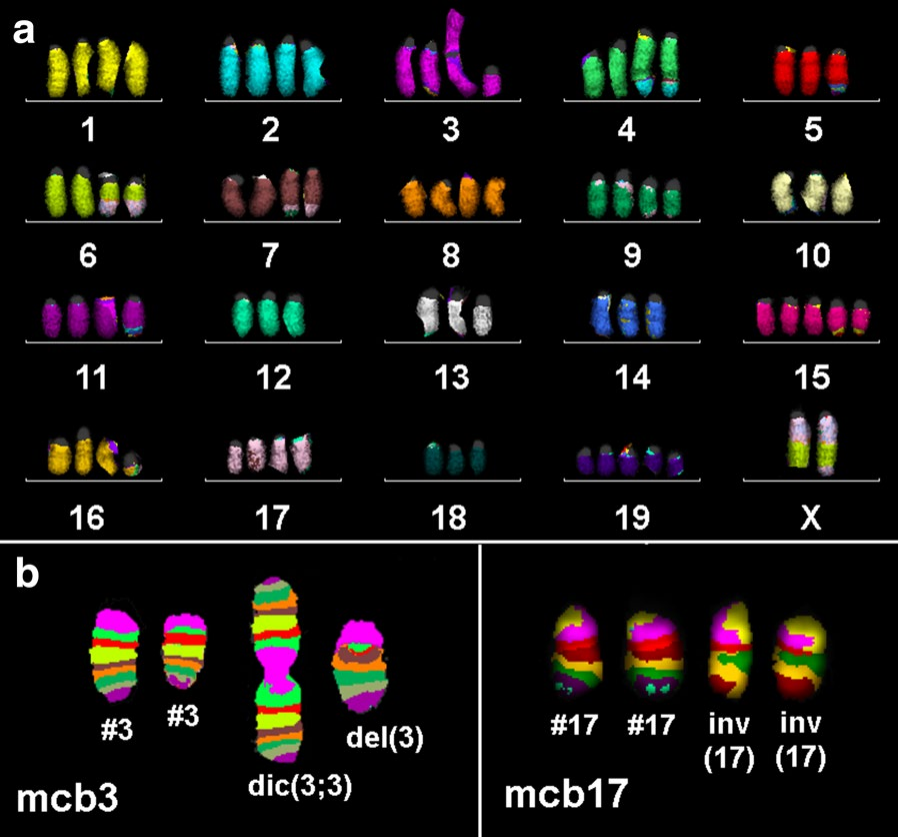
mFISH (**a**) and selected mcb results (**b**) for murine MM cell line AB22. For mcb3 two normal chromosomes (#3), a dic(3;3)(A1;A1) (dic(3;3)) and a del(3)(A3F1) (del(3)) are depicted. Also application of mcb17 revealed the presence of two normal chromosomes 17 (#17) and two chromosomes 17 with inversion inv(17)(CE5) (inv(17))

The tumor cell line AB22 was near tetraploid (Fig. 2a)—here the composite karyotype: 73~79<4n>,-X,-X,der(X)t(X;6)(C~D;C1),der(X)(XA1→XC~D::6C1→6G2::XF1→Xqter),dic(3;3)(A1;A1),del(3)(A3F1),der(4)(4A1→4C3::4C3::4C5→4C7::4C7→4C5::2F3→2qter),der(4)(4A1→4C3::4C3::4C5→4C7::4C7→4C5::2F3→2qter),-5,der(5)t(5;11)(G2;D~E),der(6)t(X;6)(D;C1),der(6)t(X;6)(D;C1),der(7)t(7;9)(F4;F1),der(7)t(7;9)(F4;F1),-10,-12,-13,-14,der(15)t(5;15)(G2;E),der(15)t(5;15)(G2;E),der(15)(15pter→15E::6D→6E::15E→15qter),+der(15)(15A1→15E::6D→6E::15E→15qter),+der(15)(15pter→15E::6D→6E::15E→15qter),del(16)(B2),der(16)(pter→B2::B1->qter),inv(17)(CE5),inv(17)(CE5),-18,+19.

**Fig. 3:**
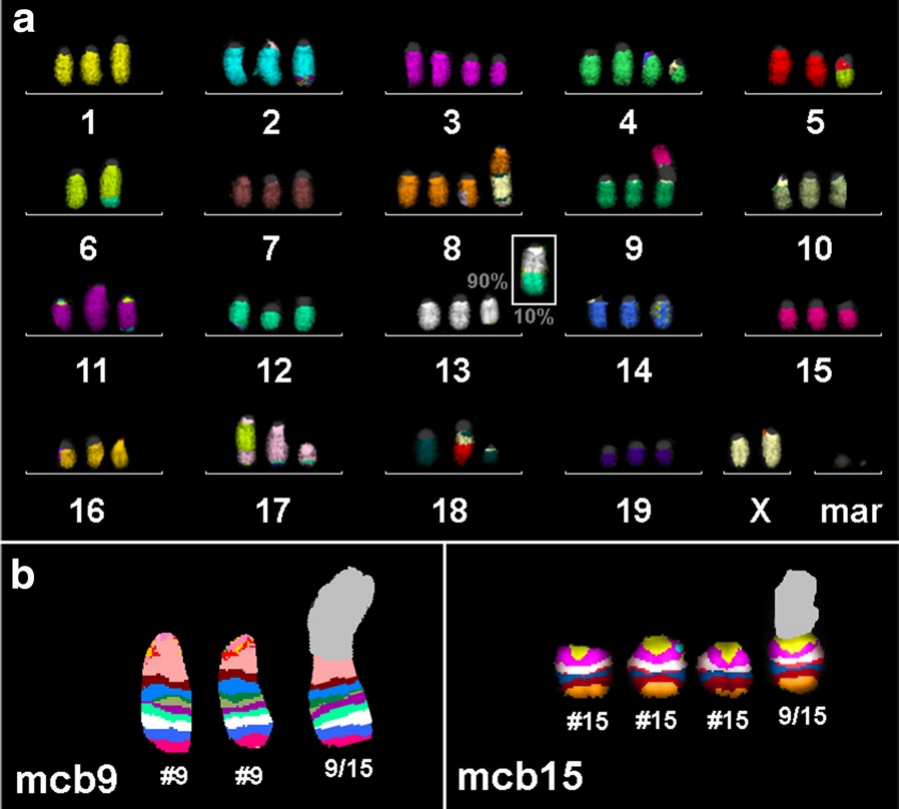
**a** mFISH result of the main clone being present in 90% of the cell line AC29 is shown here. The only difference in the subclone comprising 10% of the cells is that the del(13)(A5B) is replaced by a der(13)t(13)t(12;13) as shown in white square. **b** Result for mcb9 and 15 highlight the dic(9;15)(A1;A1) here labeled as 9/15

Here examples for the mcb characterization of dic(3;3)(A1;A1) and del(3)(A3F1) by mcb3 and of inv(17)(CE5)x2 are shown in Fig. 3b.

#### Ac29

AC29 turned out to be hyper-triploid with one main clone (90%) and one smaller subclone (10%). The main clone karyotype (Fig. 3a) had the following karyotype: 63<3n>,X,-X,der(X)(pter→A1::A2→qter),der(1)(pter→D::E4→G::H2→qter),der(1)(pter→C5::C2→qter),del(2)(E2E5),der(2)(2pter→2H3::19C3→19D2::11D→11qter),+del(3)(A3E3),+del(3)(A3E3),+del(4)(C4),der(5)t(5;6)(B;B3),-6,der(6)t(6;12)(G1;C2),+der(8)(8pter→8A3::8B3→8E2::18D→18E4::1E4→1G::18D→18E4::1E4→1G::18E4→18D::1E4→1G),dic(9;15)(A1;A1),del(11)(B4E1),der(11)(pter→B4::A2→qter),der(11)t(2;11)(H3;D),der(12)t(6;12)(G1;C2),del(13)(A5B),der(17)t(11;17)(D;E5),der(17)(17pter→17B~C::6B1→6G3::6B1→6G3::17B~C→17E5::2H3→2qter),der(17)(17pter→17E5::17E4→17E5::11E1→11qter),der(18)t(5;18)(B1;D3),del(18)(B1C),+mar1,+mar2.

The subclone was just characterized by a translocation between chromosome 12 and 13 {der(13)t(13)t(12;13)} instead of del(13)(A5B) compared to the main clone (Fig. 3a).

As examples for mcb the characterization of the dic(9;15)(A1;A1) is shown in Fig. 3b.

Two marker chromosomes could be resolved here, neither by multicolor fluorescence in situ hybridization using all 21 murine whole chromosome paints as probes (mFISH—Fig. 3a) nor by mcb. Thus, most likely they are derivatives of the centromere-near region of any of the murine chromosomes—subband A1, which do not specifically stain by any euchromatic DNA-probe. Accordingly, the marker chromosomes could be left overs of the dic(9;15)(A1;A1) and a del(?)(A1).

### aCGH

**Fig. 4:**
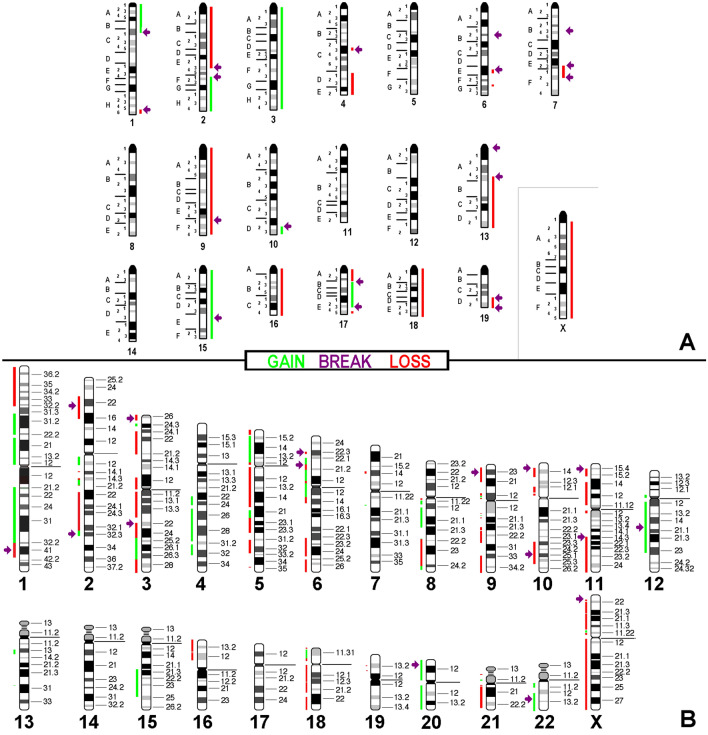
aCGH results for cell line AB1. In **a** copy number variations detected are summarized with respect to a tetraploid karyotype. Gains are depicted as green bars (one more copy = light green; two more copies = dark green), loss of one copy is depicted as a red bar and loss of two copies is depicted as a dark-red bar. Breaks are registered here as arrows. In **b** results of in silico translation for AB1 to human genome are shown the same way as in **a**

**Fig. 5:**
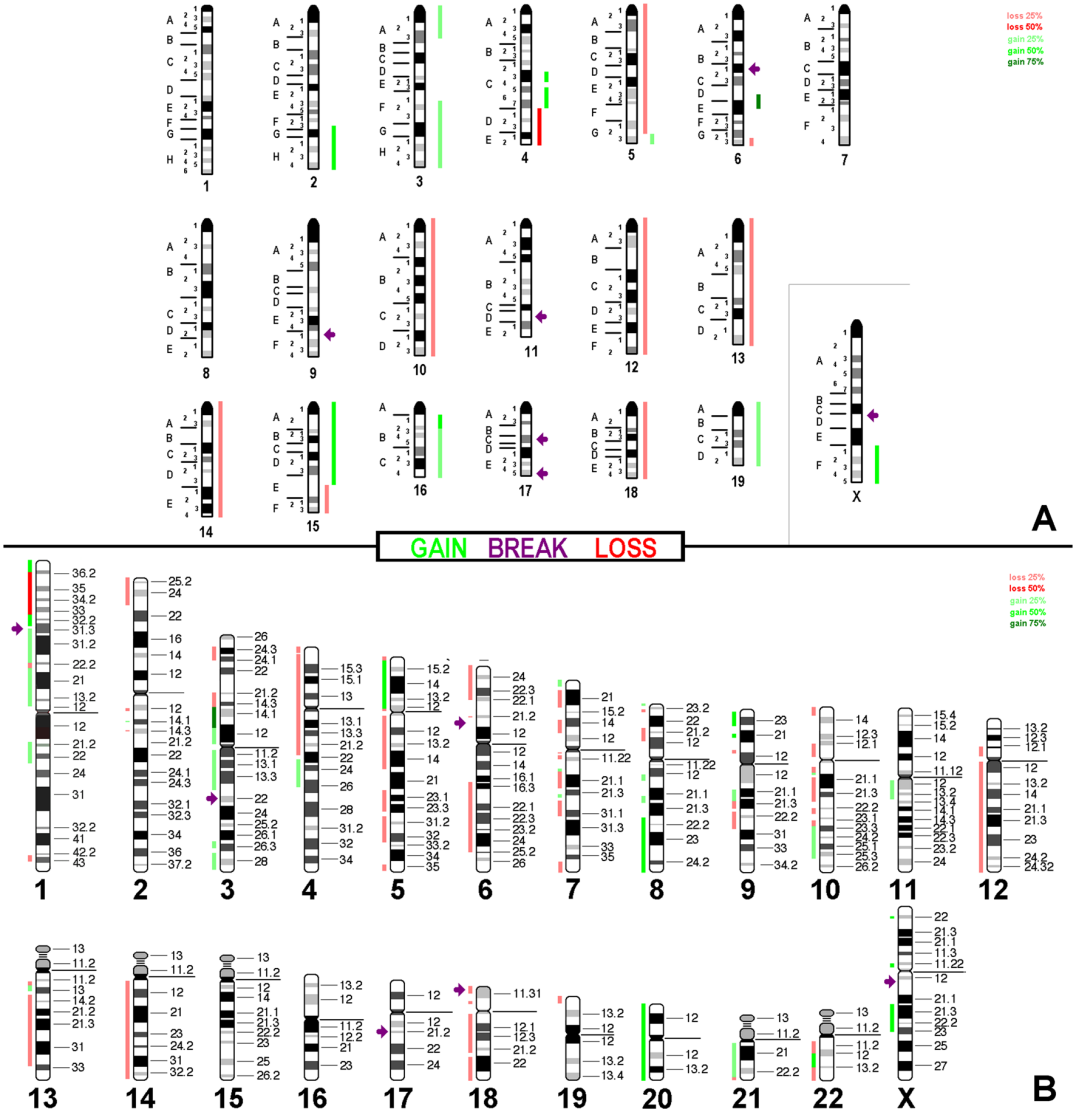
aCGH results for cell line AB22 depicted with respect to a tetraploid karyotype; legend like in Fig. 4

**Fig. 6:**
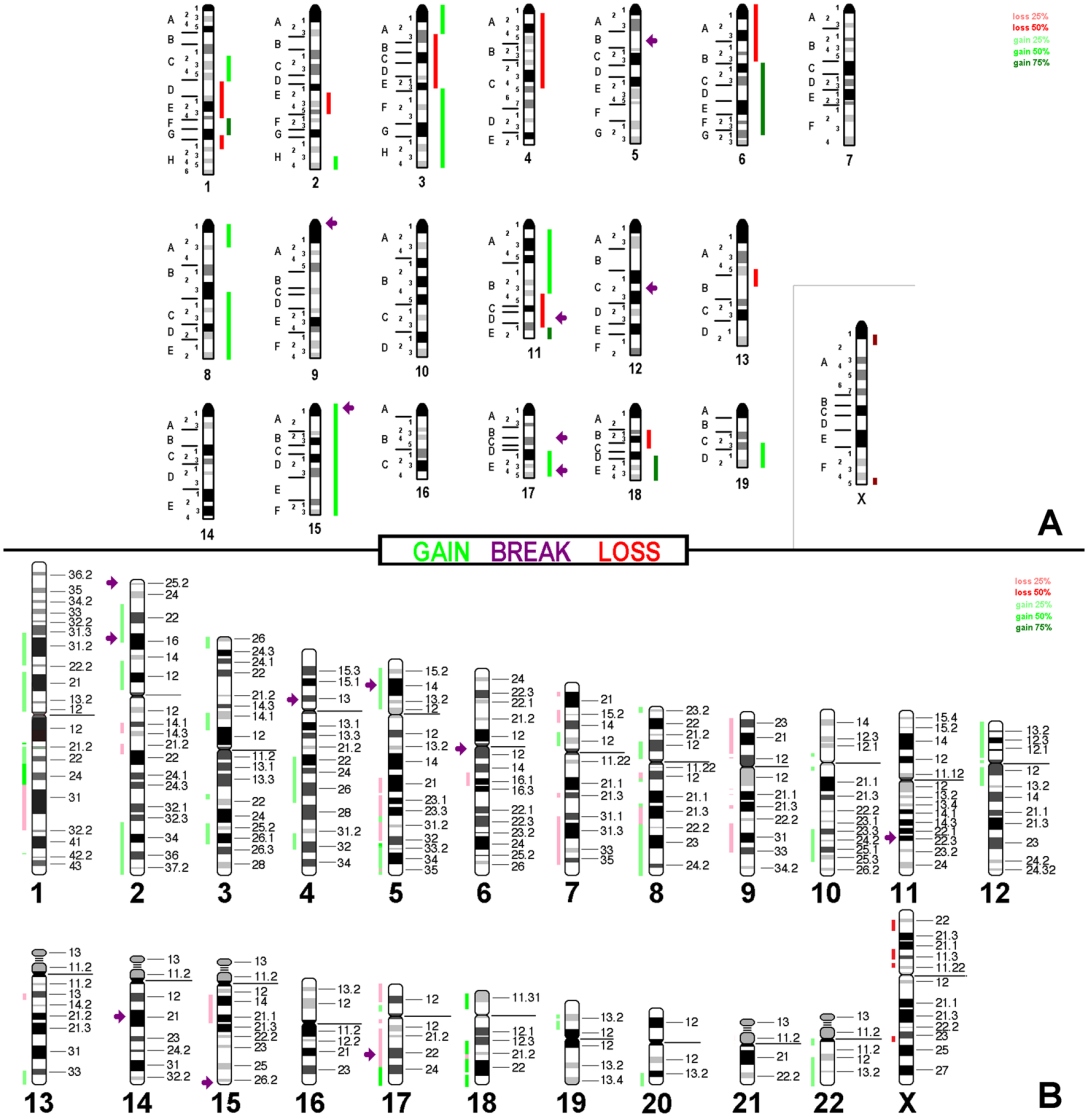
aCGH results for cell line AC29 depicted with respect to a triploid karyotype; legend like in Fig. 4

Array comparative genomic hybridization (aCGH) data (Additional file 1: Table 1) together with which FISH results could be summarized in Figs. 4a, 5a and 6a. These results were translated to the corresponding homologous regions in the human genome as depicted in Figs. 4b, 5b and 6b. All in the evaluation included imbalances were larger than 3.5 mega base pairs.

### Data-analyses

The common aberrations and cytogenetic changes that frequently occur in MM [22] revealed for all three cell lines to be less similar to human biphasic MM subtype (Table 1). According to Table 1, AB1 has 14/16 (88%) aberrations in common with human epithelioid and 17/21 (81%) aberrations with human sarcomatoid MMs. For AB22 it was 18/20 (94%) and 17/21 (81%) concordance to human epithelioid and sarcomatoid MMs, respectively. And for AC29 similarities of human epithelioid or sarcomatoid MMs was 15/20 (75%) versus 15/22 (68%). Thus, all three cell lines seemed to be best suited as models for human epithelioid MMs.

**Table 1:** Comparison of ‘translated’ imbalances of murine MM-cellines AB1, AB22 and AC29 with human epitheloid, biphasic and sarcomatoid MM, according to Krismann et al. [22]

CNV detected in cell lines AB1	Epithelial MM	Biphasic MM	Sarcomatoid MM
del(1)(pter32)	?	+	(+)
amp(1)(p31q32)	+	−	(+)
del(2)(p23p16)	+	−	−
amp(2)(q12q21.2)	(+)	−	+
del(2)(q22q32)	−	−	?
del(3)(p22p10)	?	−	+
del(3)(q10q24)	?	−	−
amp(3)(q25q26)	?	?	+
del(3)(q27qter)	?	?	−
amp(5)(p15p12)	+	−	+
del(5)(p12q15)	?	?	+
amp(6)(p22.1q12)	(+)	(+)	(+)
del(6)(q22.3qter)	+	+	+
amp(8)(q11.2q21.2)	+	(+)	+
del(9)(pterqter)	+	+	+
del(10)(p15p12)	+	+	+
del(10)(q23q25)	(+)	(+)	(+)
del(11)(pterp10)	?	−	+
del(11)(p10qter)	?	+	+
amp(15)(q21.2q24)	+	+	?
del(18)(p11.2qter)	+	?	+
amp(20)(pterqter)	+	−	(+)
del(21)(q11.1qter)	−	?	?
del(X)(pterqter)	+	−	−
Sum for +	11/16	6/19	12/21
Sum for (+)	3/16	3/19	5/21
Sum for + and (+)	14/16	9/19	17/21
CNV detected in cell lines AB22	Epithelial MM	Biphasic MM	Sarcomatoid MM
del(1)(pter32)	+	+	(+)
amp(1)(p32p10)	+	−	+
del(3)(p24p24)	+	−	(+)
del(3)(p21.2p14.2)	?	?	(+)
amp(3)(p14.2q21)	+	+	+
del(3)(q26qter)	?	?	−
del(4)(pterq21.1)	+	+	+
amp(5)(p15.3p12)	+	−	+
del(5)(q11qter)	?	?	(+)
del(6)(q16q25)	+	+	+
amp(7)(pterp22)	+	+	?
del(7)(p22qter)	−	−	+
del(8)(p22p12)	+	+	+
amp(8)(q22qter)	+	(+)	+
del(9)(q21.2q22.3)	(+)	(+)	?
del(10)(pterq23.2)	+	+	+
amp(11)(q11q13.3)	?	−	?
del(12)(p12.1qter)	?	−	+
del(13)(q13q32)	+	+	+
del(14)(q11qter)	+	+	+
del(18)(pterqter)	+	(+)	+
del(19)(pterp13.3)	?	(+)	(+)
amp(20)(pterqter)	+	−	(+)
del(22)(q11.2q11.2)	+	+	+
del(22)(q13.2qter)	+	+	+
Sum for +	17/20	11/22	15/23
Sum for (+)	1/20	4/22	6/23
Sum for + and (+)	18/20	15/22	21/23
CNV detected in cell lines AC29	Epithelial MM	Biphasic MM	Sarcomatoid MM
amp(1)(p31 q25)	−	−	(+)
amp(2)(p23p11.2)	−	−	+
amp(2)(q33qter)	+	−	+
amp(3)(pterq24.3)	+	−	−
amp(3)(p14.1p13)	+	+	?
amp(3)(q25q26.2)	(+)	(+)	+
amp(5)(p15.2p13.1)	+	−	+
del(5)(q21q32)	?	?	(+)
amp(5)(q33qter)	?	+	−
del(6)(q15q16.1)	+	+	+
del(7)(p21p15.1)	+	+	?
amp(7)(p13p11.1)	−	−	−
del(7)(q21.3q36)	+	+	+
amp(8)(p12p11.1)	+	−	−
amp(8)(q22.2qter)	+	(+)	+
del(9)(pterq33)	+	+	+
del(13)(q13q14.1)	+	+	+
del(15)(q13q21.1)	−	−	(+)
del(17)(pterp12)	+	+	(+)
del(17)(q11q23)	?	−	−
amp(17)(q24qter)	+	−	(+)
amp(19)(p13.2p13.1)	?	−	−
amp(20)(q13.2qter)	+	−	+
del(X)(p22p11.2)	(+)	−	−
Sum for +	14/20	8/23	10/22
Sum for (+)	1/20	2/23	5/22
Sum for + and (+)	15/20	10/23	15/22

Only imbalances present in any of the three human MMs are listed

CNV = copy number variation; + = aberration present in the cell line, − absent in the cell line; ? = no clear correlation possible, as it can be + or − in human cases

Also in Table 2 region, where four tumor suppressor genes meant to play important role in human MM are localized, were checked for copy number variant presence in the three studied murine MM cell lines. No correlations were found here.

**Table 2:** The four tumor suppressor genes most often involved in human MM acc. to [7–16], being deleted and/or mutated there are compared for copy number variant observed in the three studied cell lines

Human gene/murine homologue	AB1	AB22	AC29
*BAP1* deletion 3p21.1	(+)	+	No CNV
*CDKN2A* deletion 9p21.2	No CNV	No CNV	No CNV
*TP53* deletion 17p13.1	No CNV	No CNV	No CNV
*NF2* deletion 22q12.2	No CNV	No CNV	No CNV

Genomic locations (HG19/GRCh37): *BAP1* - chr3:52,435,020-52,444,121; *CDKN2A* - chr9:21,967,751-21,994,490; *TP53* - chr17:7,571,720-7,590,868; *NF2* - chr22:29,999,545-30,094,589

+ = partially deleted; (+) = possibly deleted; no CNV = no copy number alteration; dup = duplication instead of deletion

## Conclusions

The murine MM cell lines AB1, AB22 and AC29 were studied in this paper for the first time by molecular cytogenetics combined with aCGH. This enabled to determine their genetic alterations and imbalances and align these with human MMs. mFISH using whole chromosome painting probes revealed the general characteristics of the cell lines, like the ploidy, clonal and nonclonal changes as well as numerical and intrachromosomal structural aberrations. By mcb interchromosomal alterations as duplications, deletions or inversions, and chromosomal breakpoints involved could be uncovered, as previously reported [23–28]. The aCGH data was aligned with the FISH results and also used to determine breakpoints of unbalanced rearrangements (Additional file 1: Table 1).

The cell lines A1 and AC29 were tetraploid; as in both cell lines derivative chromosomes were present twice, it is possible that polyploidization was a result of cell culture, and tetraploidy was absent in original tumor. Such so-called telomere-driven tetraploidization in the context of cell culture-related factors as trypsin treatment, increasing number of cell-culture passages, and oxygen exposure [29, 30] was discussed before. However, as no karyotype of tumor or early cell passages of A1 and AC29 are available, this is just speculation and cannot be tested by any means.

Interestingly, a deletion of *CDKN2A* gene is considered as one of the most typical alterations in human MMs [7–9]. In the AB1 and AC29 there was indeed a deletion in the murine homologous region; however in cell line AB22 this region was duplicated (Additional file 1). For other tumor suppressor genes *BAP1*, *NF2* and *TP53* thought to play important roles in human MMs [10–16], there is even less or no concordance in the copy number variant regions of the three cell lines (Additional file 1).

Nonetheless, the overall similarities of copy number variants found in the three murine MM cell lines compared to human MM are striking. A shown in Table 1 all three cell lines can serve as models for human epithelioid MM. As similarities are also high for sarcomatoid MM, also here they may be used as models for. However, AB1, AB22 and AC29 are definitely not models for human biphasic MM.

## Methods

### Murine MM cell lines

The murine cell lines AB1 and AC29 were obtained from Cell Bank Australia (Westmead, Australia, order #s CBA-0144 and CBA-0152) and AB22 European Collection of Authenticated Cell Cultures (Salisbury, UK—order# ECACC 10092307). For this study, the cells were cultivated and divided into two portions, worked up cytogenetically (portion 1), and used to extract whole-genomic DNA (portion 2) as previously described [24].

### Molecular cytogenetics

Fluorescence in situ hybridization (FISH) was performed as previously described [24]. “SkyPaintTM DNA Kit M-10 for Mouse Chromosomes” (Applied Spectral Imaging, Edingen-Neckarhausen, Germany) was used for multicolor-FISH (mFISH) applying whole chromosome paints, and murine chromosome-specific multicolor banding (mcb) probe mixes for FISH-banding [31]. At least 30 metaphases were acquired and analyzed for each probe set on a Zeiss Axioplan microscope, equipped with ISIS software (MetaSystems, Altlussheim, Germany). Array-based comparative genomic hybridization (aCGH) was completed according to standard procedures with “SurePrint G3 Mouse CGH Microarray, 4x180K” (Agilent Technologies, Santa Clara, CA, USA).

### Data analysis and translation

The regions of imbalances and breakpoints in AB1, AB22 and AC29 were characterized after analyses of aCGH and mcb data, and aligned with their human homologous regions using Ensembl Genome Browser, as previously described [24]. The data we obtained was compared with the literature [22] (Tables 1 and 2).

## Supplementary information

**Additional file 1: Table 1.** The regions of gain and loss of copy numbers, as well of breakpoints of balanced rearrangements, observed in AB1, AB22 and AC29 and the corresponding homologue regions in humans, are listed as cytoband and position (GRCh37/hg19).

## Data Availability

All data generated or analysed during this study are included in this published article and its supplementary information files.

## Abbreviations

aCGH: Array comparative genomic hybridization
*BAP1*: Breast cancer associated gene 1 (*BRCA1*) associated protein 1 gene
*CDKN2A*: Cyclin-dependent kinase inhibitor 2A gene
FISH: Fluorescence in situ hybridization
mFISH: Multicolor fluorescence in situ hybridization using all 21 murine whole chromosome paints as probes
MM: Malignant mesothelioma
mcb: Murine multicolor banding
*NF2*: Neurofibromatosis type 2 gene
*TP53*: Tumorsuppressorprotein 53 gene
