# Supplementary material for: Cytogenomic characterization of three murine malignant mesothelioma tumor cell lines
Source: Mol Cytogenet. 2020 Sep 9;13:43. doi: 10.1186/s13039-020-00511-4 (PMC7488062; doi:10.1186/s13039-020-00511-4)
Supplement: Supplementary file 1 — Additional file 1: Table 1. The regions of gain and loss of copy numbers, as well of breakpoints of balanced rearrangements, observed in AB1, AB22 and AC29 and the corresponding homologue regions in humans, are listed as cytoband and position (GRCh37/hg19). [file 13039_2020_511_MOESM1_ESM.docx]

**Suppl. Table 1**

The regions of gain and loss of copy numbers, as well of breakpoints of balanced rearrangements, observed in AB1, AB22 and AC29 and the corresponding homologue regions in humans, are listed as cytoband and position (GRCh37/hg19).

| **A) AB1 Cell line** | | | |  |
| --- | --- | --- | --- | --- |
| **Region** | **Gain** | **Homologue Region in Human** | |  |
|  |  | **Cytoband** | **Position (GRCh37/hg19)** |  |
| 1A1-C1 | 1x | 8q11.21-q12.1  8q13.1-q21.11  6p12.3-p12.2  6q11-q13  6p12.1-p11.2  2q14.3-q21.1  2q11.2-q12.2  13q33.1  2q32.1-q32.2  2q32.2-q37.3 | 8:50767106-56535248  8:67336477-76107163  6:49796129-52568703  6:61967179-73920868  6:56223874-58686221  2:128848553-131914911  2:97151065-106819719  13:103237605-103533914  2:189007277-190504466  2:190506076-242812118 |  |
| 2F-H4 | 1x | 20p13-p11.21  20p13  20q11.21-q13.32  20q13.32-q13.33 | 20:1736101-25606620  20:102147-1447942  20:29933153-58056214  20:58148222-62907435 |  |
| 3A1-H4 | 1x | 8q21.11-q21.3  8q12.3-q13.1  3q24-q25.1  3q26.2-q26.32  3q26.32-q27.1  4q27-q31.1  9p11.2  21p11.2  9p12  9q13  13q13.2-q14.11  3q25.1-q26.2  4q31.23-q32.2  1q21.1-q23.1  1p22.1-p12  4q26  4q22.3-q26  1p31.3-p22.2  7q11.23 | 8:76197937-87035414  8:64075897-67315825  3:148467535-148965303  3:167857105-178105807  3:178137517-182818465  4:122242382-141190230  9:45446787-46098069  21:10369840-10592667  9:42028291-42246766  9:68139917-68294733  13:34463185-41254213  3:149055816-167822106  4:150966383-163096512  1:144676687-158154741  1:93905157-120696915  4:119596924-120703320  4:95284699-119338945  1:68589539-89738135  7:76282730-76575579 |  |
| 10D2-D3 | 1x | 12q13.2-q23.3 | 12:55351591-104351507 |  |
| 15A1-F3 | 2x | 5p15.31-p12  8q22.1-q24.3  22q12.3-q13.33  12p11.1  12q12-q13.2 | 5:8927745-42888975  8:97446632-146158346  22:35962951-51222438  12:33476533-34210697  12:38607141-55072925 |  |
| 17B1-E3 | 1x | 6p22.1-p21.32  6p21.2-p12.3  3p25.1-p24.3  2q12.2-q12.3  19p13.3  5q21.1-q22.1  18p11.32-p11.22 | 6:29322703-33297218  6:39266498-49681826  3:16307846-20231899  2:107383985-108798215  19:4229082-6862967  5:102759315-110063021  18:2534401-9972541 |  |
| **Region** | **Loss** | **Homologue Region in Human** | |  |
|  |  | **Cytoband** | **Position (GRCh37/hg19)** |  |
| 1H5-H6 | 1x | 1q32.2-q42.13 | 1:207575939-227644727 |  |
| 2A1-E3 | 1x | 2p12.1-13  2q14.1  9q34.11-34.3  9q33.2-34.1  2q22.1-32.1  11q12.1  11p11.2-14.2  10p13-15.1 | 10:15427950-26868173  2:112974505-113379867  9:128309435-138124704  9:120763799-128299267  2:139292421-187530602  11:55313107-57986386  11:26188819-48637160  10:5873489-15414078 |  |
| 4C3 | 1x | 9p24.1-p21.2 | 9:6847129-27220407 |  |
| 4D1-E2 | 1x | 1p36.33-p32.2~1 | 1:894315-59012766 |  |
| 6E2-E3 | 2x | 3p26.3-p25.2 | 3:61304-12897767 |  |
| 6F3-G1 | 2x | 12p13.31-p11.21 | 12:9901365-30943693 |  |
| 7E3-F2 | 1x | 11p15.4-p15.1  16p13.11  16p13.11-p12.3  16p12.3-p12.2 | 11:3631069-17360027  16:15260325-15369270  16:16681590-18325190  16:18608156-21351663 |  |
| 9A1-F4 | 1x | 11q14.3-q22.3  19p13.2  7p14.3-p14.2  11q22.3-q25  15q21.2  15q21.2-q25.1  6p12.2-p12.1  6q13-q14.3  15q25.1  3q22.3-q24  3q22.1-q22.3  3p21.31-p21.1  3p24.1-p22.2  3p22.2-p21.31 | 11:89860533-107436639  19:8919008-11689880  7:33134362-36494039  11:107452617-134843539  15:51349622-51942502  15:51961808-78956872  6:52656530-55784577  6:74104388-86360515  15:79042978-80196839  3:138372654-148087492  3:129931635-138353358  3:46446256-52346387  3:27753690-37261140  3:37269243-46423369 |  |
| 13A5-D2 | 1x | 6p23-p22.3  9q22.1-q22.32  5q35.2-q35.3  5q31.1-q31.2  9q21.32-q21.33  9q22.32-q22.33  9p13.1  9q12-q13  9p11.2  8q22.1  5p15.33-p15.31  5q14.3-q15  5q13.2-q14.3  5q11.1-q13.2  1p11.2  5p12 | 6:15104709-20060798  9:91031851-97067712  5:173750964-177039611  5:134073478-137090938  9:86231955-90340399  9:97320957-99417669  9:38810965-40707569  9:65585614-65901647  9:43623473-43941731  8:97247028-97373828  5:191425-7935441  5:84566270-96144383  5:70265557-84371909  5:49569996-68922426  1:121149401-121350677  5:43446298-46118514 |  |
| 16A1-C4 | 1x | 16p13.3-p13.11  16p13.11  8q11.21  12p11.21  22q11.21  3q27.1-q29  3q29  3q11.1-q21.2  3p12.3-p11.1  21q11.2-q22.3  21q11.2  18p11.21  2q21.1 | 16:3283710-15197331  16:15478874-16187414  8:48206338-49865275  12:32634919-33054761  22:19010381-22338262  3:182965714-195325931  3:195428230-197771581  3:93527487-125343459  3:75865702-90309600  21:15515528-43438088  21:14535253-14714360  18:15016525-15155234  2:132604281-132757591 |  |
| 17A1-B1 | 1x | 6q25.2-q25.3  6q27  6q25.3-q27  6q27  5q15-q21.1  16p13.3  5q35.1  6p21.32-p21.2  21q22.3  19p13.12  19p13.2 | 6:155053083-160101646  6:167120855-167552070  6:160103032-166797236  6:167859539-170893754  5:96202316-98405239  16:222880-3208490  5:171946752-172722349  6:33359177-39058058  21:43490502-45122943  19:15270296-15808207  19:8366687-8811037 |  |
| 17E5 | 1x | 2p23.2-p16.3  2p16.3-p16.2  18p11.32 | 2:29033520-51699597  2:51709987-53282184  18:861722-2534400 |  |
| 18A1-E4 | 1x | 10p11.21  10p12.1-p11.22  10p12.1  10p11.21  18p11.32  18q11.1-q12.3  2q14.3  5q22.1-q22.2  5q31.2-q32  5q22.2-q23.3  5q32-q33.1  18p11.22-p11.21  18q21.31-q21.32  18p11.21  18q12.3-q21.31  18q22.1-q23 | 10:35284099-35521818  10:28950711-32678701  10:27747786-28722506  10:35676708-37094546  18:112543-599224  18:18528605-41073893  2:127805408-128786667  5:110280120-112296881  5:137225085-147624774  5:112310736-130339352  5:147647374-150177176  18:10202644-11518916  18:54267924-58201586  18:11649353-13871680  18:41355914-54244819  18:66339761-78010601 |  |
| 19D1-D3 | 2x | 10q23.2-q26.11 | 10:87474356-119459995 |  |
| XA1.1-F5 | 2x | Xp21.1-p11.23  Xq23-q24  Xq24-q27.1  Xq27.2-q28  Xq23  Xp22.31-p22.2  Xp22.11-p21.1  Xq11.1-q23  Yp11.2  Xp11.22-p11.21  Xp22.2  Xp22.2-p22.11 | X:37364439-47520178  X:115210308-117585111  X:117586665-140073167  X:140429142-154494231  X:114569624-114885545  X:8784578-9687806  X:23850309-37316857  X:62853720-114517895  Y:4132374-5642381  X:52987493-56318562  X:9688235-9917528  X:10415591-23849592 |  |
| **Region** | **Breakpoint** | **Homologue Region in Human** | |  |
|  |  | **Cytoband** | **potential tumor associated genes** |  |
| 1C1 | t | 2q32.3 | 2:192542794-192553251 (*OBFC2A*) |  |
| 1H5 | t | 1q41 | 1:223394161-223537544 (*SUSD4*) |  |
| 2E3 | t | 11p14.1 | 11:28129795-28355054 (*METTL15*) |  |
| 2F | t | 20p13 | 20:3229951-3388272 (*C20orf194*) |  |
| 4C3 | del | 9p23 | 9:8314246-10612723 (*PTPRD*) |  |
| 6E2 | del/ inv | 3p26.1 | 3:6811688-7783215 (*GRM7*) |  |
| 7E3 | t | 11p15.4 | 11:4935900-4936922 (*OR51G2*) |  |
| 7B1B2 | t | n.a. | n.a. |  |
| 9F1 | t | 3q22.1 | 3:131100515-131107674 (*NUDT16*) |  |
| 10D2 | del | 12q15 | 12:71031853-71314623 (*PTPRR*) |  |
| 13A5 | del | 6p22.3 | 6:16299343-16761722 (*ATXN1*) |  |
| 13A1 | dic | 10p15.1 | 10:5807186-5884095 (*GDI2*) |  |
| 15E1 | t | 22q13.1 | 22:40806285-41032706 (*MKL1*) |  |
| 17B1 | t | 6p21.32 | 6:32595956-32614839 (*HLA-DQA1*) |  |
| 17E3 | del/ inv | 2p22.1 | 2:39963200-40006407 (*THUMPD2*) |  |
| 19D1 | del/ t | 10q25.1 | 10:105889646-105992120 (*WDR96*) |  |
| 19D3 | t | Xp22.33 | X:1455509-1501578 (*IL3RA*) |  |
| **B) AB22 Cell line** | | | |  |
| **Region** | **Gain** | **Homologue Region in Human** | |  |
|  |  | **Cytoband** | **Position (GRCh37/hg19)** |  |
| 2F1-qter | 2x | 20p13-p11.2 20pter-p13 20q11.2-q13.2 20q13.2-qter | chr20:1755455-25625984 chr20:0-1467297 chr20:31301793-59481159 chr20:59573167-64276082 |  |
| 3A1-A3 | 1x | 8q21.13-q21.2 8q12.3-q13.1 3q26.2-q26.32 | chr8:75285702-86044133 chr8:63163338-66403590 chr3:168139317-175619000 |  |
| 3F1-qter | 1x | 1q21.1-q23.1 1p22.1-p11.2 4q22.3-q26 1p31.3-p22.2 | chr1:147100246-158184951 chr1:93439600-120801119 chr4:94363548-119782165 chr1:68121446-89272452 |  |
| 4C3-C3 | 2x | 9p23-p22.3 | chr9:10000000-16000000 |  |
| 4C5-C7 | 2x | 9p21.2-p21.2 1p32.1-p31.1 1pter-p36.22 | chr9:25847129-27300708 chr1:58654679-67096416 chr1:933238-8000000 |  |
| 5G2-qter | 1x | 7q11.23-q11.23 7q22.1-q22.1 7p22.3-p22.1 7q21.3-q22.1 13q12.13-q13.2 | chr7:73145680-76520510 chr7:99955218-102551307 chr7:115497-6732018 chr7:97968996-99631744 chr13:26210757-33680968 |  |
| 6qD-qE | 3x | 3p14.2-p12.3 | chr3:66032928-75273450 |  |
| 15pter-E | 2x | 5p15.3-5p12 8q22.1-q24.23 8q24.23-qter 22q12.3-q13.2 | chr5:8927633-42888873 chr8:96434404-136853592 chr8:136870681-144619253 chr22:35566904-43270000 |  |
| 16B1-qB2 | 2x | 3q27.1-q29 | chr:183247926-195044710 |  |
| 16B2-qter | 1x | 3q29-q29 3q11.2-q21.2 3p12.3-p11.2 21q11.2-q22.3 | chr3:195044710-198044710 chr3:93808643-125624615 chr3: 75817777-90260450 chr21:14143207-42017979 |  |
| 19pter-qter | 1x | 11q12.1-q13.3 9q21.11-q21.31 2q14.1-q14.1 9pter-p24.1 10q11.23-q21.1 10q23.2-q26.11 | chr11:58108647-68942254 chr9:68221841-80162449 chr2:113413562-113564048 chr9:51702-6659223 chr10:50157843-52780322 chr10:87474356-119459995 |  |
| XF1-qter | 1x | Xq21.22-q23 Xp11.22-p11.21 Xp22.2-p22.2 Xp22.2-p22.1 | chrX:94193017-115283323 chrX:52958297-56292129 chrX:9720195-9949488 chrX:10447551-23831475 |  |
| **Region** | **Loss** | **Homologue Region in Human** | |  |
|  |  | **Cytoband** | **Position (GRCh37/hg19)** |  |
| 4C7-qter | 2x | 1p36.22-p32.2 | chr1:8000000-51547094 |  |
| 5pter-G2 | 1x | 7q11.23-q21.2 7q22.1-q22.3 7q36.1-q36.3 2p23.3-p23.2 22q12.2-q12.3 4p16.3-p16.3 4p16.3-q22.1 1p22.2-p22.1 4p16.3-4p16.3 12q24.33-12qter 22q11.23-q12.1 12q23.2-q24.1 12q24.1-q24.31 12q24.31-q24.33 7p11.2-p11.2 7q11.21-q11.22 | chr7:77072929-93047200 chr7:102691476-105569647 chr7:150896031-157417511 chr2:26171597-28801596 chr22:31626131-32115679 chr4:1115354-3874051 chr4:4183016-88079035 chr1:89498675-9336,782 chr4:559,243-1058871 chr12:131894446-132,945,956 chr22:24805798-28760295 chr12:107931580-110048615 chr12:110050988-121059734 chr12:121109639-131852016 chr7:55951659-56116445 chr7:67371788-72722850 |  |
| 6qG2-qter | 1x | 12p12.1-p11.21 | chr12:24800000-32384500 |  |
| 10pter-qter | 1x | 6q16.2-q25.2  2q12.3-q12.3  10q21.1-q22.1  22q11.22-q11.23  21q22.3-qter  19pter-p13.3  12q23.3-q23.3  22q12.3-q12.3  12q13.2-q23.3 | chr6:100098103-154676710  chr2:108449081-109621141  chr10:53435340-73103214  chr22:23054310-24635537  chr21:43939787-4666,000  chr19:281181-4172053  chr12:103965531-107783160  chr22:32387312-33076428  chr12:54962426-103957729 |  |
| 12pter-qter | 1x | 2p25.1-p23.3 2p25.1-p25.1 2pter-p25.1 7q22.3-q31.1 7p21.3-p21.1 7q31.1-q31.1 14q12-q22.1 14q23.1-q32.33 7q36.3-q36.3 7p21.1-p15.3 | chr2:10162883-26139074 chr2:9214594-10144790 chr2:172258-9138189 chr7:105569791-108131740 chr7:12522126-19709187 chr7:108131761-112496091 chr14:24687986-51784456 chr14:58199894-105939623 chr7:157432951-159145209 chr7: 19713790-22489274 |  |
| 13pter-qter | 1x | 10pter-p15.1 1q42.3-q43 7p14.2-p13 6p22.3-p22.1 6pter-p22.3 6p22.3-p22.3 9q22.1-q22.32 5q35.2-q35.3 5q31.1-31.2 9q21.32-q21.33 9q22.32-q22.33 9p13.1-p12 5pter-p15.31 5q14.3-q15 5q11.2-q14.3 5p12-p12 | chr10:92758-5823659 chr1:235166745-239921359 chr7:36484897-43566331 chr6:20064992-28544283 chr6:181261-17755386 chr6:17758154-20060567 chr9:88426259-94305430 chr5:174325424-177612610 chr5:134737788-137755249 chr9:83617040-87725484 chr9:94558675-96664521 chr9:38669564-39362991 chr5:191310-7935328 chr5:85240523-96808680 chr5:50274162-85076091 chr5:43118864-45905752 |  |
| 14pter-qter | 1x | 3p14.3-3p14.1 3p24.3-p24.1 14q22.1-q22.1 6p21.2-p21.2 10q22.2-q22.3 10q22.3-q22.3 3p21.2-14.3 3p25.1-p25.1 10q11.22-q11.23 10q23.2-q23.2 14q22.1-q23.2 14q11.2-q11.2 14q11.2-q12 13q12.12-q12.13 13q12.11-q12.11 13q14.2-q14.2 13q12.13-q12.13 13q12.12-q12.12 13q14.2-q14.3 8p23.1-p23.1 8p21.3-p12 13q14.11-q14.2 13q14.3-q33.1 | chr3:57956447-64024024 chr3:23104895-27667234 chr14:51805337-52106937 chr6:39101990-39298710 chr10:73110406-79495343 chr10:80053353-80210587 chr3:52316044-57945503 chr3:15203607-16266323 chr10:45868532-49967632 chr10:80261810-87216559 chr14:52221917-58163176 chr14:19735448-22100195 chr14:19735448-22100195 chr13:24761821-24937784 chr13:19633139-22705235 chr13: 49247854-49587268 chr13:25025580-26075099 chr13:23279259-24322217 chr13:49620101-51782351 chr8:9812600-11884194 chr8:20349073-29293682 chr13:40895805-49224923 chr13:52651898-102437231 |  |
| 15E-qter | 1x | 22q13.2-qter 12p11.21-p11.21 12q12-q13.2 | chr22:43270000-50784010 chr12:33053427-34105230 chr12: 38213339-54655672 |  |
| 18pter-qter | 1x | 10p11.21-p11.21 10p12.1-10p11.22 10p12.1-10p12.1 10p11.21-p11.21 18pter-p11.32 18q11.2-q12.3 2q14.3-q14.3 5q22.1-q22.2 5q31.2-q32 5q22.2-q23.3 5q32-q33.1 18p11.22-p11.21 18q21.31-q21.32 18p11.21-p11.21 18q12.3-q21.3 18q22.1-qter | chr10:34995171-35221367 chr10:28661782-32389773 chr10:27458857-28624637 chr10:35331990-36805618 chr18:112543-599224 chr18:20949510-43493928 chr2:126578751-128029093 chr5:110944421-112961184 chr5:137889396-148245211 chr5:112975039-131003659 chr5:148267811-150797614 chr18:10202647-11518917 chr18:56600693-60560532 chr18:11733896-13871681 chr18:43775949-56577588 chr18:68672524-80209980 |  |
| **Region** | **breakpoint** | **Homologue Region in Human** | |  |
|  |  | **Cytoband** | **potential tumor associated genes** |  |
| 6C1 | t | No Homologues |  |  |
| 9F1 | t | 3q22.1 | 3:132757235-133116636 (*Tmem108*) |  |
| 11D | t | 17q21.2 | **17:38922490-38928414 (*KRT26*)** |  |
| 17C | inv | 6p21.1 | 6:41196062-41206620 (*TREML4*) |  |
| 17E5 | inv | 18p11.32 | 18:2537524-2571508 (*METTL4*) |  |
| XC | t | Xq13.1 | X:68048840-68061990 (*EFNB1*) |  |
| **C) AC29 Cell line** | | | | |
| **Region** | **Gain** | **Homologue Region in Human** | | |
|  |  | **Cytoband** | **Position (GRCh37/hg19)** | |
| 1qC2-qC5 | 1x | 2q33.1-qter | 2:195728066-241869966 | |
| 1E4-G | 2x | Yq11.23  1p11.1  1q21.1  1q23.1-q25.3 | Y:26212371-26397883  1:121135999-121390756  1:143925313-145127773  1:158547113-182909192 | |
| 2H3-qter | 1x | 20q13.32-qter | 20:59573167-64276082 | |
| 3pter-A3 | 1x | 8q21.13-q21.2  8q12.3-q13.1 | 8:75285702-86044133  8:63163338-66403590 | |
| 3E3-qter | 1x | 3q25.1-q26.2  4q31.3-q32.1  4q32.2  1q21.2-q23.1  1p11.2-p21.3  4q22.3-q26  1p22.2-p31.1 | 3:149338029-168104318  4:150045231-160313119  4:160328589-162175360  1:147100246-158184951  1:93439600-120801119  4:94363548-119782165  1:68121446-89272452 | |
| 6B3-G1 | 1x | 12p13.33  12p13.31-p13.32  12p12.3-p13.2  12p13.31  22q11.21  10q11.21  3q21.3  3p13-p14.1  3pter-p25.2  2p11.2-p13.2  1p31.2  4q27  4q22.1-q22.3  3p25.2  3p25.1  2p13.3  2p11.2 | 12:12900-2743910  12:2793954-7543294  12:9748769-32384500  12:7905547-9061868  22:17084921-18176973  10:42782538-45674259  3:126006258-129319641  3:64032928-75273450  3:12360-12059043  2:70805311-86867996  1:67166227-67851415  4:120041924-121273532  4:88257546-94351949  3:12075139-12855873  3:12897779-15118656  2:68487905-70797831  2:88002903-88874857 | |
| 8A1-A3 | 1x | 19p13.2  13q33.1-qter  8q11.21-q11.23  8pter-p23.2  8p23.2  8p23.1-p23.2  8p11.23-p12 | 19:7112172-8069598  13:102881565-114327455  8:36859024-42639769  8:397986-5501230  8:5510625-5890510  8:5904979-6718069  8:29332949-36820056 | |
| 8B3-qter | 1x | 10p11.21-p11.22  19p13.12-p13.13  22q12.3  19p13.11 | 10:32752983-34863341  19:12627694-14572196  22:33262346-35459147  19:16052230-19664128 | |
| 11A2-B3 | 1x | 22q12.1-q12.2  7p12.1-p13  7p11.2-p12.1  2p13.3-p16.2  5q35.2  5q33.2-35.1  5q31.1  5q33.1-q33.2  5q35.5  1q42.13  17p11.2 | 22:28772603-31626130  7:43924481-53156756  7:53191518-55250238  2:53660668-68467594  5:173309722-174284250  5:154952277-172505309  5:131159221-134727937  5:151002149-154951429  5:178104362-180869289  1:227732052-228515511  17:17013944-21429010 | |
| 11E1-qter | 2x | 17q24.2-qter | 17:68228066-83227287 | |
| 15pter-qter | 1x | 5p12-p15.31  8q22.1-q24.23  8q24.23-qter  22q12.3-qter  12q12-q13.2  12p11.1 | 5:8927633-42888873  8:96434404-136853592  8:136870681-144619253  22:35566904-50784010  12:38213339-54655672  12:33053427-34105230 | |
| 17E1-E5 | 1x | 2p p16.2-23.2  18p11.22-p11.32 | 2:28810654-53031720  18:2534402-9972544 | |
| 18D-qter | 2x | 18q22.1-qter  18q12.3-q21.31  18p11.21  18q21.31-q21.32  18p11.21-p11.22  5q32-q33.1 | 18:68672524-80209980  18:43775949-56577588  18:11733896-13871681  18:56600693-60560532  18:10202647-11518917  5:148267811-150797614 | |
| 19C3-qter | 1x | 10q22.3-q26.11 | 10:87474356-119459995 | |
| **Region** | **Loss** | **Homologue Region in Human** | | |
|  |  | **Cytoband** | **Position (GRCh37/hg19)** | |
| 1D-E4 | 1x | 2q14.3  2q14.1-q14.2  18q2.32-q22.1  5q21.1-q21.2  Yq11.23  2q21.2-q22-1  1q25.3-32.2 | 2:121828372-125590121  2:113678530-121820449  18:60684670-67661356  5:99084032-103392710  Y:26212371-26397883  2:132380816-137850173  1:182909192-207361619 | |
| 1G-H2 | 1x | 23.2-32.13 | 1:158547113-207361619 | |
| 2E2-E5 | 1x | 15q13.3-q21.2 | 15:32614079-51005976 | |
| 3A3-E3 | 1x | 9p11.2  9p13  9q21.11  21p11.2  13q13.2-q14.11 | 9:39883273-40101748  9:63544183-63698999  9:66274930-66379174  21:9860388-10114639  13:33906643-40680076 | |
| 4A1-C4 | 1x | 8q12.1-q12.3  8q21.3-q22.1  8q12.3  6q15-q16.2  9q13.1-q21.2  9q22.33-q33.2  9q21.31-q21.33  9p21.2-p24.1 | 8:55737745-61783006  8:86044134-96234554  8:62182367-63105957  6:87084169-99797137  9:27325075-38472102  9:97275612-120726664  9:80378606-83600221  9:26847129-27300708 | |
| 6pter-B3 | 1x | 7p21.3  7q21.3  7q31.1-q32.1  7p14.3-p15.3  7q32.1-q36.1 | 7:7093365-12492920  7:93101327-97872805  7:112498864-128461586  7:23214537-33063634  7:128671928-149886174 | |
| 11B3-E1 | 1x | 17q24.1-q24.2  17q23.2-q24.1  7q32.1  17q21.31-q21.32  17q12-q21.31  17q21.32-q23.2  17q11.2-q12  17q11.1-q12  17p13.2-pter  17p12-13.2 | 17:64952801-68114549  17:62298466-64764269  7:128466497-128616266  17:45629380-47073225  17:38195870-45561456  17:47482968-62248837  17:30731359-37831987  17:27198624-30526883  17:143647-4628746  17:4630159-15722490 | |
| 13A5-B | 1x | 9q22.1-q22.32  5q35.1-q35.2  5q31.1-q31.2  9q21.32-q21.33 | 9:88426259-94305430  5:174325424-177612610  5:134737788-137755249  9:83617040-87725484 | |
| 18B1-C | 1x | 5q31.2-q32  5q22.2-q23.3 | 5:137889396-148245211  5:112975039-131003659 | |
| XA1-A2 | 2x | Xp11.22-p11.23  Xp11.4-p11.23  Xp 23-p24 | X:48455475-51616130  X:37491877-47660779  X:115988254-118451148 | |
| XF4-qter | 2x | Xp22.2-p22.11 | X:10447551-23831475 | |
| **Region** | **breakpoint** | **Homologue Region in Human** | | |
|  |  | **Cytoband** | **potential tumor associated genes** | |
| 5B | t | 4p13.3 | 9:35609530-35646807 (*CD72*) | |
| 9A1 | t | 11q22.3 | 11:106555148-106889250 (*GUCY1A2*) | |
| 11D | t | 17q21.31 | 17:41322511-41363706 (*NBR1*) | |
| 12C2 | t | 14q21.2 | 14:47120222-47121028 (*RPL10L*) | |
| 15A1 | t | 5p14.1 | 5:26880709-27121257 (*CDH9*) | |
| 17B | ins | No homologues |  | |
| 17E4 | inv | 2p16.3 | 2:48844937-48960287(*GTF2A1L*) | |
| 7C | t | 15q26.3 | 15:101459420-101610317 (*LRRK1*) | |
| 12A2 | t | 2p25.3 | 2:3192696-3381653 (*TSSC1*) | |
| 13D2 | t | 5q11 | 5:49961733-50142356 (*PARP8*) | |
